# The novel HSV-1 U_S_5-1 RNA is transcribed off a domain encoding U_S _5, U_S _4, U_S _3, U_S _2 and α22

**DOI:** 10.1186/1743-422X-7-103

**Published:** 2010-05-21

**Authors:** Vladimir Jovasevic, Bernard Roizman

**Affiliations:** 1 The Marjorie B. Kovler Viral Oncology Laboratories, The University of Chicago, Chicago IL 60637, USA

## Abstract

**Background:**

The genome of herpes simplex virus 1 encodes at least 84 transcripts from which proteins are translated and several additional RNAs whose status as mRNAs is unknown. These RNAs include latency-associated transcript, Ori_S_1 and Ori_S_2 RNAs and in case of α4 null mutant additional transcript that spans the junction between L and S component of the HSV-1 genome. Current data do not suggest that a peptide is translated from these RNAs.

**Results:**

We describe here a novel RNA designated U_S_5-1 that spans 4.5 kb of the unique-short (U_S_) region. The RNA initiates in U_S_5 and terminates in the α22 open reading frame. It is expressed antisense to U_S_5, U_S_4, U_S_3 and ICP22 mRNAs. This transcript is expressed with γ_2 _kinetics and has a half-life of 80 minutes.

**Conclusion:**

These results identify a novel transcript encoded within HSV-1 genome. Since no major hypothetical open-reading frames are present in this transcript it is feasible that this RNA exerts its function as a non-coding RNA.

## Background

The original report of the organization of the unique short (U_S_) DNA sequences of the herpes simplex virus 1 (HSV-1) listed 12 open reading frames (ORFs) designated U_S_1 (α22) through U_S_12 (α47) [1]. Subsequent studies led to the discovery of three additional transcripts and at least two non coding transcripts. The coding transcripts were U_S_8.5 mRNA co-terminal with U_S_8 and U_S_9 [2], U_S_1.5 co-terminal with the α22 transcript [3], and U_S_3.5 co-terminal with the U_S_3 mRNA [4]. The non coding transcript Ori_S_1 initiated in α22 or α47 ORFs, run across the origins of DNA synthesis and terminated at the transcription termination site of the α4 gene. Ori_S_2 terminated at or near the transcription initiation site of the α22 or α47 gene [5]. Latency-associated transcript (LAT) is the only viral transcript detected in latently infected neurons [6]. Although several potential ORFs can be found within LAT sequence, none of them are transcribed in the context of viral infection [7,8]. In addition, Schaffer and colleagues [9] reported transcripts in cells infected with Δα4 mutant that spanned the junction between the L and S components of HSV-1 DNA. While it is suspected that LAT plays a role in the establishment and maintenance of latent infection, its function is not well established. Also, the roles of Ori_S _transcripts or Δα4-specific transcript are unknown. In this report we describe an additional long transcript designated U_S_5-1 RNA. As the designation implies, this transcript originates in the U_S_5 ORF and terminates in the α22 ORF.

## Results

### RNA antisense to U_S_3 is expressed in cells infected with wild-type virus

**Figure 1:**
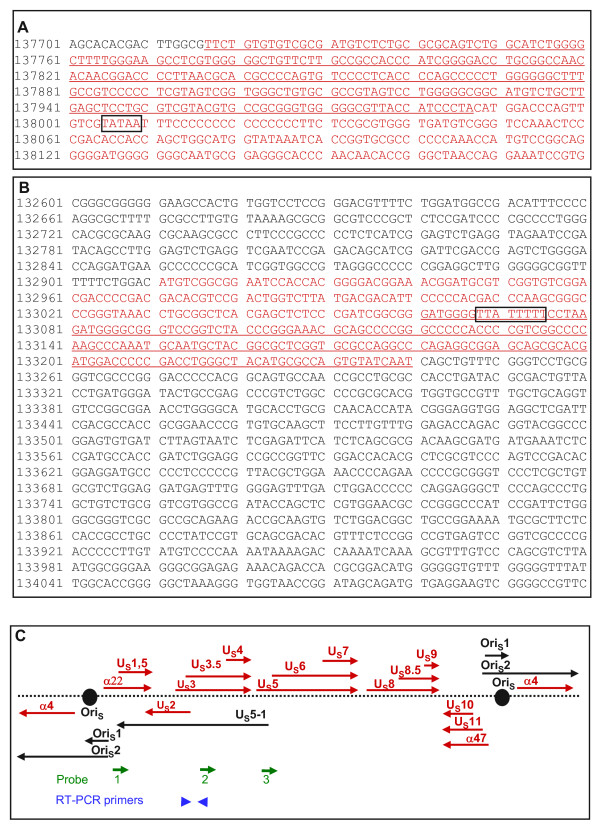
**Genomic location of the U_S_5-1 RNA**. (A) In red is shown the sequence of the probe 3, used for the determination of the 5'-end of the U_S_5-1 RNA. Underlined are first 270 bases of the probe 3, which was the size of the protected fragment in the RNase protection experiment to determine the 5'-end of the U_S_5-1 RNA. In the box is the sequence of potential TATA box. (B) In red is shown the sequence of the probe 1, used for the determination of the 3'-end of the U_S_5-1 RNA. Underlined are the last 180 bases of the probe 1, which was the size of the protected fragment. Sequence of a possible polyadenylation signal for the U_S_5-1 transcript is shown in the box. (C) Location and the direction of transcription of RNAs expressed from the unique-short region are represented by arrows. Red arrows represent protein-coding RNAs, black arrows represent non-coding RNAs. Location and the direction of the transcription of the single-stranded RNA probes used in the experiments described in this report are represented by green arrows. Primers used for RT-PCR detection of U_S_5-1 transcript are shown in blue arrowheads.

**Figure 2:**
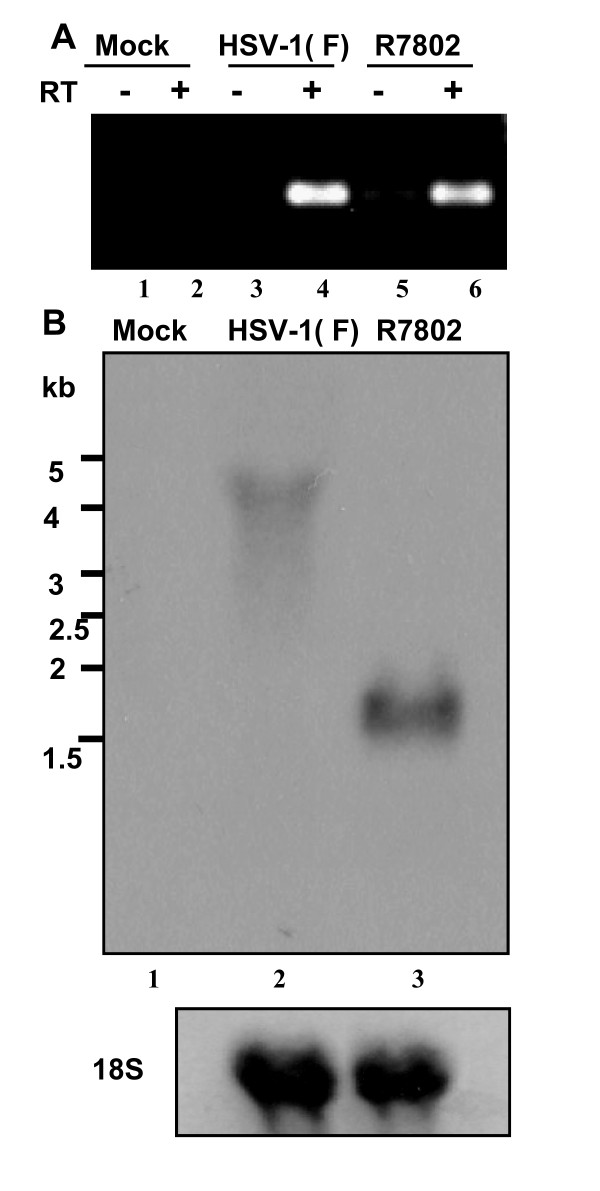
**A RNA antisense to U_S_3 accumulates in cells infected with wild-type virus**. (A) Cultures of RSC in 25 cm^2 ^flasks were mock infected (lanes 1 and 2) or infected with 10 PFU of HSV-1(F) (lanes 3 and 4) or R7802 (lanes 5 and 6) per cell. Total RNA was extracted 18 h later and reverse transcription was performed on 1 μg of total RNA. As a control, 1 μg of RNA was subjected to the same treatment, except that reverse transcriptase was omitted (lanes 1, 3, and 5). (B) Cultures of RSC in 25 cm^2 ^flasks were either mock infected (lane 1) or infected with 10 PFU of HSV-1(F) (lane 2) or R7802 (lane 3) per cell. Total RNA was extracted 18 h later and aliquots of 10 μg of total RNA were separated on 1% formaldehyde agarose gel, transferred to Biodyne B nylon membrane and hybridized with single-stranded RNA probe 2. For the size determination molecular weight marker positions are shown. The amount of 18S ribosomal RNA is shown as the loading control.

The experiments reported here resulted from the observation that a mutant derived from R7208 contained an unexplained 1.6 kb RNA antisense to the U_S_3 ORF. Briefly, in this mutant the α22 gene was deleted and in the course of studies of a selected isolate of this mutant we found that it contained an insertion containing a stop codon upstream of the U_S_3 ORF. To determine whether RNA antisense to U_S_3 is expressed in cells infected with wild-type virus, rabbit skin cells (RSC) were exposed to 10 PFU of HSV-1(F) per cell, RNA was extracted 18 h later and subjected to RT-PCR or Northern blot analysis. For RT-PCR analysis cDNA was generated from total RNA using primer 51(R), specific for RNAs antisense to U_S_3. PCR reaction was performed using the 51(F) as forward and 51(R) as reverse primer. While reverse transcription with 51(R) primer would also generate cDNA from U_S_2 transcript, this cDNA would not be amplified under PCR reaction used in this experiment since 51(F) primer lies upstream from U_S_2 transcript (Fig. 1C). Results of RT-PCR analysis (Fig. 2A) show that antisense RNA was also expressed in cells infected with wild-type virus. Northern blot analysis, performed using 1% agarose gel, (Fig. 2B) verified these results and additionally indicated that in cells infected with the wild type virus the RNA was approximately 4.5 kb long.

### The U_S_5-1 transcript spans genomic region between U_S_5 and α22

**Figure 3:**
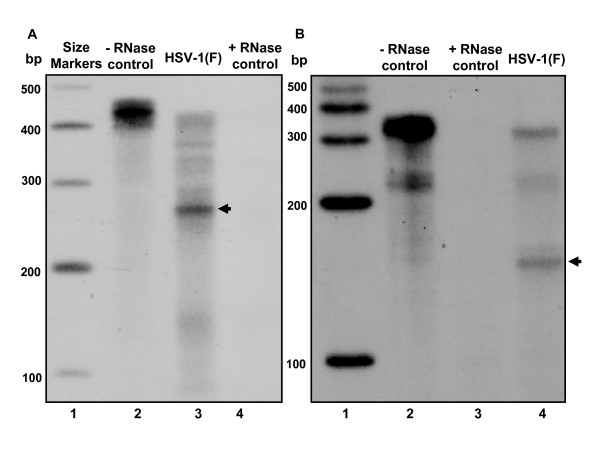
**The U_S_5-1 RNA spans genomic region between U_S_5 and ICP22**. Cultures of RSC in 25 cm^2 ^flasks were infected with 10 PFU of HSV-1(F) and total RNA was extracted 18 h after infection. The 3'- and 5'-ends were determined by RNase protection assay. In addition to samples from infected cells (panel A, lane 3 and panel B, lane 4), included were control samples of the equivalent amount of yeast total RNA, untreated (panel A, lane 2 and panel B, lane 2) or treated with RNAseA/RNaseT1 mix (panel A, lane 4 and panel B, lane 3). Single-stranded RNA marker was included for size determination (panels A and B, lane 1).

Experiments were next carried out to determine the region of the S component spanned by the U_S_5-1 mRNA. To determine the location of the 5'-end we relied initially on observations carried out on the R7802 recombinant virus. In cells infected with this mutant, the truncated U_S_5-1 RNA terminated at the polyadenylation signal inserted into the U_S_2/U_S_3 boundary (V. Jovasevic. and B. Roizman, manuscript in preparation). Knowing the size of this RNA (~1.6 kb), we estimated the location of its 5'-end on the basis of the assumption that the sequences upstream of the insertion site would be identical in both R7802 and wild-type virus. In order to fine map the location of the 5'-end of the U_S_5-1 transcript, we performed a series of northern blot analyses that helped us place the 5'-end within the sequences covered by the probe 3 (data not shown). To determine more precisely the location of the 5'-end of the U_S_5-1 transcript we performed an RNase-protection assay using probe 3. The results of this experiment reveal the presence of a protected fragment with size of about 270 bases (Fig. 3A, arrow). In figure 1A the sequence of the probe 3 is shown in red and underlined are the first 270 bases of the probe 3 protected in the RNase protection assay. A potential TATA box sequence can be observed upstream from the last underlined base (boxed sequence), making it more likely that the underlined sequence of the probe 3 is the 5' terminus of the U_S_5-1 transcript.

On the basis of the size of the U_S_5-1 transcript and the location of its 5'-end we estimated the location of the 3'-end to be within the ICP22 ORF. To determine more precisely the location of the 3'-end, RSC were infected with 10 PFU of HSV-1(F) per cell, RNA extracted 18 h later and subjected to RNase protection assay using a probe that spanned the predicted termination site identified within ICP22 ORF (probe 1). The probe 1 protected fragment of approximately 180 bases long (Fig. 3B, arrow). In figure 1B the sequence of the probe 1 is shown in red and underlined are the last 180 bases of the probe 1 protected in the RNase protection assay. In the vicinity of the identified 3'-end we observed a sequence that could potentially serve as a polyadenylation sequence for the U_S_5-1 transcript (boxed sequence). In addition to the 180 bases long fragment we also observed another protected fragment that was equal in length to the full-length probe. This fragment does not correspond to the U_S_5-1 transcript, considering that the U_S_5-1 transcript is ~4.5 kb long. It is possible that a different RNA, expressed antisense to ICP22, is present in infected cells.

Figure 1C illustrates schematically the domains of transcripts mapped in the S component to date. Single-stranded RNA probes used for Northern blot and RNase protection assay are represented by green arrows. Primers used for RT-PCR analysis are represented by blue arrowheads.

### The U_S_5-1 transcript is expressed with γ_2 _kinetics

**Figure 4:**
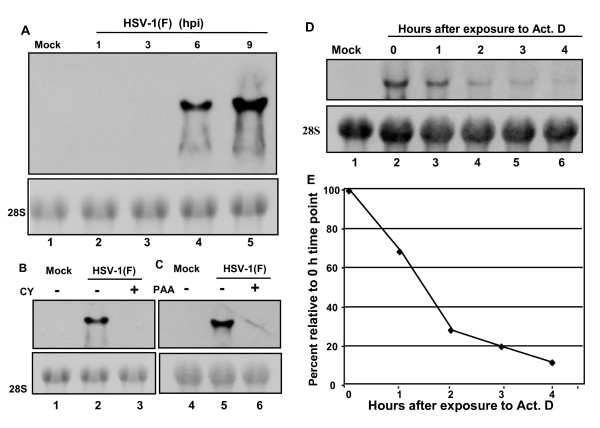
**Kinetics and stability of U_S_5-1 RNA**. (A) Cultures of RSC in 25 cm^2 ^were mock infected (lane 1) or infected with 10 PFU of HSV-1(F) per cell and total RNA was extracted 1 (lane 2), 3 (lane 3), 6 (lane 4) or 9 h (lane 5) after infection and analyzes for the expression of U_S_5-1 by northern blot using 0.7% formaldehyde agarose gel. The 28S ribosomal RNA is shown as the loading control. (B) Cultures of RSC in 25 cm^2 ^flasks were mock infected (lane 1) or infected with 10 PFU of HSV-1(F) per cell in the absence (lane 2) or presence (lane 3) of 100 μg/ml cycloheximide. Total RNA was extracted 9 h after infection and analyzed for the expression of U_S_5-1 by northern using 0.7% formaldehyde agarose gel. The 28S ribosomal RNA is shown as the loading control. (C) Cultures of RSC in 25 cm^2 ^flasks were mock infected (lane 1) or infected with 10 PFU of HSV-1 (F) per cell in the absence (lane 2) or presence (lane 3) of 300 μg/ml of phosphonoacetate (PAA). Total RNA was extracted 9 h after infection and analyzed for the expression of U_S_5-1 by northern blot using 0.7% formaldehyde agarose gel. The 28S ribosomal RNA is shown as the loading control. (D) Cultures of RSC in 25 cm^2 ^flasks were mock infected (lane 1) or infected with 10 PFU of HSV-1(F) per cell. At 6 h after infection actinomycin D (Act. D) was added to the concentration of 100 μg/ml of medium. RNA was extracted 0 (lane 2), 1 (lane 3), 2 (lane 4), 3 (lane 5) or 4 h (lane 6) after the addition of Act. D and analyzed for the expression of U_S_5-1 by northern blot using 0.7% formaldehyde agarose gel. The 28S ribosomal RNA is shown as the loading control. (E) The amount of the U_S_5-1 transcript in each experimental group in panel D was determined by measuring the density of each band on the membrane by UN-SCAN-IT densitometer (Silk Scientific, Inc., Orhem, UT). Relative amount of U_S_5-1 transcript, expressed as the percent of the U_S_5-1 transcript at time zero, was plotted as the function of time.

In the experiments described next, we evaluated the timing and requirements for the expression of the U_S_5-1 RNA. In the first series of experiments, RSC were exposed to 10 PFU of HSV-1(F) per cell, RNA extracted 1, 3, 6, or 9 h later and subjected to northern blot analysis using probe 2 for detection of the U_S_5-1 transcript. The results (figure 4A) show that the U_S_5-1 could not be detected at 1 or 3 h after infection. It was first detected at 6 h after infection.

In the next series of experiments we determined whether the onset of synthesis of U_S_5-1 requires *de novo *viral protein synthesis. RSC were infected with 10 PFU of HSV-1(F) per cell in the presence or absence of 100 μg/ml cycloheximide. Total RNA was extracted 9 h later and subjected to northern blot analysis. Addition of cycloheximide completely abrogated the expression of the U_S_5-1 transcript (Fig. 4B), indicating that *de novo *protein synthesis is essential for its expression.

The purpose of the next series of experiments was to determine whether viral DNA synthesis was required for the synthesis of U_S_5-1 RNA. RSC were exposed to 10 PFU of HSV-1(F) per cell in the presence or absence of 300 μg of PAA per ml. Total RNA was extracted 9 h later and subjected to northern blot analysis. PAA completely abrogated the expression of the U_S_5-1 transcript (Fig. 4C), indicating that the replication of viral DNA is essential for its expression, and therefore that the U_S_5-1 transcript is expressed with γ kinetics. Since the expression of the RNA was completely abrogated in the presence of PAA and not just diminished we conclude that the U_S_5-1 transcript is expressed with γ_2_kinetics.

### The U_S_5-1 transcript has a half-life of about 80 minutes

In the final set of experiments we proceeded to evaluate the stability of the U_S_5-1 transcript. RSC were infected with 10 PFU of HSV-1(F) per cell. At 6 h after infection the cells were exposed to 100 μg of actinomycin D per ml of medium. The RNA was extracted at 0, 1, 2, 3 or 4 h after the addition of the actinomycin D and subjected to northern blot analysis (Fig. 4D). The intensity of individual bands was measured by a densitometer and plotted as a function of time (Fig. 4E). The results shown in figure 4E indicate that the half-life of the U_S_5-1 transcript was approximately 80 min.

## Discussion

Relevant to this report are the following:

(i) One possible explanation for the failure to detect the 4.5 kb RNA in earlier studies is the failure of transfer of high molecular weight RNA from gels containing high concentrations of agarose. In the studies reported here, we detected the 4.5 kb RNA on transfer from gels containing 1%, or less, of agarose (Fig. 2C), but not following transfer from gel containing 1.2% agarose (data not shown).

(ii). The U_S_5-1 RNA is dependent on viral DNA synthesis for its accumulation. In contrast, the Ori_S_1 RNA does not require *de novo *viral protein or DNA synthesis for its accumulation [10]. The measured half-life (80 min.) is in the line with that of other HSV-1 RNAs reported to be between 60 and 150 minutes regardless of the kinetic group to which a gene belongs [11].

(iii). In principle, viruses evolve continuously and do not retain DNA sequences that have no functions related to their survival in nature. The function of U_S_5-1 RNA is not known. The U_S_5-1 RNA contains only two large ORFs of 300+ codons. An ORF of approximately the same size is present in the corresponding sequences of HSV-2 DNA. However, In HSV-1 DNA the first large ORF initiates with the fifth methionine codon and its predicted amino acid sequences is not conserved in the corresponding HSV-2 ORF. We cannot exclude the possibility that the very small ORFs upstream of the large one are expressed. It seems it would be very unlikely that an RNA 4.5 kb long would encode a peptide with less than 100 amino acids, as it would be highly energy inefficient. However, it would not be unprecedented, since Drosophila's tal gene expresses 1.5 kb long transcript that codes for several 11 amino acid long peptides [12]. Overall, the data do not currently support the hypothesis that the U_S_5-1 directs protein synthesis.

(iv) As noted earlier, the accumulation of HSV-1 non coding RNAs is not unprecedented. There is considerable current interest in non coding RNAs inasmuch as they frequently serve as precursors of microRNAs or directly regulate the expression of genes located antisense to the RNA. We should note however that none of the studies published to date reported microRNA derived from the domain of the U_S_5-1 RNA.

## Conclusion

In this report we identified a novel HSV-1 transcript, expressed from the unique-short region of the viral genome. It spans the region from the U_S_5 to the α22 genes and is transcribed antisense to U_S_5, U_S_4, U_S_3 and α22 mRNAs, with γ2 kinetics. Our data suggest that U_S_5-1 is a long non-coding RNA. The role of this transcript is currently unknown, but it is plausible that, similarly to other long non-coding RNAs, it is involved in the regulation of expression of viral genes.

## Methods

### Cells and viruses

Rabbit skin cells (RSC) were originally obtained from J. McClaren. The cells were maintained in Dulbecco's modified Eagle's medium (DMEM) supplemented with 5% fetal bovine serum. HSV-1(F) is the prototype HSV-1 strain used in the laboratory [13]. Mutant virus R7802, which has the deletion of the entire ICP22 ORF has been described elsewhere [14].

### Northern blot

Cultures of RSC in 25-cm^2 ^flasks were either mock infected or infected with 10 PFU of virus per cell and maintained at 37°C in medium 199 V consisting of a mixture of 199 supplemented with 1% calf serum. The cells were harvested at time points after infection specified for each experiment. In some experiments cells were infected in the presence of 100 μg/ml cycloheximide or 300 μg/ml phosphonoacetate (PAA). Total RNA was extracted by using Trizol reagent (Invitrogen, Carlsbad, CA). Aliquots of 10 μg of total RNA were separated on 1,2%, 1% or 0.7% formaldehyde agarose gels, transferred to Biodyne B nylon membranes (Thermo Science, Rockford, IL) and hybridized with single-stranded RNA probe transcribed *in vitro *and labeled with biotin using MAXIscript kit (Ambion, Austin, TX) from DNA template driven under the T7 promoter. DNA template was generated by PCR from the HSV-1 genomic DNA. Primers used for the generation of the DNA template were the following:

Probe 2(F): TAATACGACTCACTATAGGCATATACTAGCGCGGATGCCGCGGAC

Probe 2(R): CATGCCAGTCACCAGCTTGGCCATGGTCGA

PCR reaction was performed using Pfu DNA polymerase (Stratagene, La Jolla, CA) under the following conditions: 2 min. at 94°C followed by 30 cycles of 30 s at 94°C, 30 s at 60°C, 1 min at 68°C, with final 7 min at 68°C. The location of the probe 2 within the unique-short region of the HSV-1 genome and the direction of its transcription is shown in figure 1C.

### RNase Protection assay

RNA samples were prepared as described for northern blot experiments. RNase protection assay was performed using RPA III kit (Ambion, Austin, TX) according to manufacturer's instructions. Briefly, aliquots of 10 μg of total RNA were mixed with 2 × 10^4 ^cpm of probe labeled with ^32^P-UTP using MAXIscript protocol (Ambion, Austin, TX) according to manufacturer's instructions and precipitated. RNA samples were dissolved in hybridization buffer, incubated at 56°C over night and treated with RNaseA/RnaseT1 mix (1:150 dilution) for 30 minutes. RNA was precipitated, dissolved in loading buffer, separated on 5% acrylamide/8 M urea gel and exposed to Kodak blue-bio autoradiography film (Denville Scientific, Metuchen, NJ). DNA templates from which the probe 1 for identification of the 3'-end or probe 3 for identification of the 5'-end of the U_S_5-1 RNA were transcribed were generated by PCR from the HSV-1 genomic DNA using primers for probe 1:

P1(F): TAATACGACTCACTATAGGCATATACTTTCTGTGTGTCGCGATGT

P1(R): AGTTTGTACACGGATTTCCTGGTTAG

and for probe 3:

P3(F): TAATACGACTCACTATAGGCATATACATGTCGGCGGAATCCACC

P3(R): AGTTTGTAATTGATACACTGGCGCAT for probe 3

For the PCR reactions Pfu DNA polymerase was used (Stratagene, La Jolla, CA) under the following conditions: 2 min. at 94°C followed by 30 cycles of 30 s at 94°C, 30 s at 60°C, 1 min at 68°C, with final 7 min at 68°C. The location of the probe 1 and probe 3 within the unique-short region of the HSV-1 genome and the direction of their transcription is shown in figure 1C.

### Reverse-transcription PCR

RNA samples were prepared as described for northern blot experiments. Reverse transcription was performed on 1 μg of total RNA using Reverse Transcription System (Promega, Madison, WI) according to manufacturer's instructions. Primer used for reverse transcription was 51(R): ATGTACGGAAACCAGGACTACC. As a control, the same amount of RNA was subjected to the same treatment, except that reverse transcriptase was omitted. For PCR amplification the following primers were used: 51(R) and 51(F): TCCCGGCTGCGTCGTCGTATAC

All PCR reactions were performed using Pfu DNA polymerase (Stratagene, La Jolla, CA) under the following conditions: 2 min. at 94°C followed by 30 cycles of 30 s at 94°C, 30 s at 60°C, 1 min at 72°C, with final 7 min at 72°C. PCR products were separated on 1% agarose gel and visualized by ethidium-bromide staining. The location of primers used for RT-PCR within the unique-short region of the HSV-1 genome is shown in figure 1C.

### Half-life studies

Cultures of RSC in 25-cm^2 ^flasks were either mock infected or infected with 10 PFU of virus per cell and 6 h after infection actinomycin D (AcD) was added to the concentration of 100 μg/ml. RNA was extracted 1, 2, 3 or 4 h after the addition of AcD as processed for northern blot analysis. The intensity of the bands on the film was measured by UN-SCAN-IT densitometer (Silk Scientific, Inc., Orhem, UT) and relative amount calculated as the percentage of RNA present at the time of the addition of AcD was plotted as the function of time.

## Competing interests

The authors declare that they have no competing interests.

## Authors' contributions

VJ performed the experiments and drafted the manuscript. BR reviewed and revised the manuscript.
